# Identification of a novel MIPEP splice variant with altered substrate-binding properties

**DOI:** 10.1016/j.bbrep.2025.102329

**Published:** 2025-10-29

**Authors:** Yuina Otani, Michiya Kawarai, Masaki Kobayashi, Yuka Nozaki, Rio Uchida, Kyo Tezuka, Miku Yokoyama, Yuhei Mizunoe, Takumi Narita, Hiroshi Haeno, Kanako Miyano, Yasuhito Uezono, Yoshikazu Higami

**Affiliations:** a Laboratory of Molecular Pathology and Metabolic Disease, Faculty of Pharmaceutical Sciences, Tokyo University of Science, 6-3-1 Niijuku, Katsushika-ku, Tokyo, 125-8585, Japan; b Department of Nutrition and Food Science, Graduate School of Humanities and Sciences, Ochanomizu University, 2-1-1 Otsuka, Bunkyo-ku, Tokyo, 112-8610, Japan; c Institute for Human Life Science, Ochanomizu University, Japan; d Clinical Research Support Center Mie University Hospital, 2-174 Edobashi, Tsu, Mie, 514-8507, Japan; e Research Institute for Biomedical Sciences (RIBS), Tokyo University of Science, 2641 Yamazaki, Noda, Chiba, 278-8510, Japan; f Laboratory of Pharmacotherapeutics, Faculty of Pharmacy, Juntendo University, Urayasu, Chiba, 279-0013, Japan; g Department of Pain Control Research, The Jikei University School of Medicine, Tokyo, 105-8461, Japan; h Supportive and Palliative Care Research Support Office, National Cancer Center Hospital East, Chiba, 277-8577, Japan

**Keywords:** Mitochondrial intermediate peptidase, Splice variant, Processing, ΔMIPEP, AlphaFold2

## Abstract

Mitochondrial intermediate peptidase (MIPEP) is a mitochondrial signal peptidase that removes N-terminal amino acids from mitochondrial matrix proteins. We have identified a novel *Mipep* splice variant that lacks exons 15 and 16, which we termed “ΔMIPEP”. We characterized the molecular features of ΔMIPEP by investigating its expression level in numerous mouse tissues and by performing a computer simulation that allows the prediction of protein structures and substrate-binding properties. *ΔMipep* mRNA was detected in all mouse tissues examined but at much lower levels than full-length *Mipep*. Structure prediction and docking simulation of full-length MIPEP and ΔMIPEP with substrates of MIPEP, such as malate dehydrogenase 2 (MDH2) and cytochrome *c* oxidase subunit 4, showed that entry of these substrates into ΔMIPEP with a low binding energy was greatly restricted. To determine levels of MIPEP substrates in the presence or absence of full-length MIPEP or ΔMIPEP, we created *Mipep* and *ΔMipep* overexpression 3T3-L1 cells and *Mipep* knockout (KO) cells. Western blotting showed that in *Mipep* KO cells *Mipep* overexpression slightly decreased the molecular weight of MDH2 and Sirtuin 3, another MIPEP substrate, whereas ΔMipep overexpression did not. These results indicate that ΔMIPEP fails to recognize MIPEP substrate proteins. Together, our findings indicate that ΔMIPEP is a novel splice variant that can contribute to mitochondrial signal peptidase-mediated regulation of mitochondrial protein homeostasis.

## Highlights

• Δ*Mipep*, a *Mipep* splice variant that lacks exons 15 and 16, expresses in mice tissues.
• ∙The substrate binding pocket entrance of ΔMIPEP is narrower than full-length MIPEP.
• ∙ΔMIPEP exhibits reduced substrate-binding affinity.

## 1 Introduction

Mitochondria in eukaryotic cells originated from specialized aerobic bacteria. Eukaryotic cells use ATP synthesized by glycolysis in the cytosol and by the mitochondrial respiratory chain. Most mitochondrial proteins, including tricarboxylic acid cycle and respiratory chain complexes, are encoded by nuclear genes and are synthesized in the cytosol. These proteins have N-terminal mitochondrial targeting sequences that allow entry into mitochondria through the translocase of the outer membrane/translocase of the inner membrane complex [1]. After mitochondrial proteins translocate into mitochondria, their targeting sequences are cleaved off by mitochondrial signal peptidase (MtSPase) to release mature mitochondrial proteins [2].

Mitochondrial intermediate peptidase (MIPEP) is an MtSPase that is localized in the mitochondrial matrix. MIPEP was first identified as a mammalian ortholog of yeast octapeptidyl aminopeptidase 1 (Oct1), which removes eight amino acid residues from the N-terminal region of several mitochondrial proteins [3,4]. MIPEP targets mitochondrial proteins whose N-terminal signal sequences have already been removed by mitochondrial processing peptidase (MPP). The known MIPEP substrates in yeast include malate dehydrogenase 1 (MDH1, the mammalian ortholog of which is MDH2) and cytochrome *c* oxidase subunit 4 (COX4) [[3], [4], [5]]. MIPEP has three zinc-binding sites in its active site, which support metallopeptidase activity [6].

We previously reported that caloric restriction, which has been demonstrated to exert various beneficial effects, upregulates the levels of both *Mipep* mRNA and MIPEP protein in white adipose tissues (WAT) of mice. Caloric restriction also increases the protein levels of MIPEP substrates, COX4 and MDH2 [7,8]. Moreover, we demonstrated that MIPEP contributes to the maturation of Sirtuin 3 (SIRT3), a mitochondrial deacetylase that regulates various mitochondrial functions [7]. We recently revealed that adipocyte-specific *Mipep* knockout (aMKO) mice exhibit disordered mitochondrial proteostasis and impaired activity of MIPEP substrate proteins in adipose tissues [9]. Taken together, MIPEP maintains mitochondrial quality by promoting the maturation and activation of mitochondrial proteins.

Recently, in the process of cloning *Mipep* cDNAs from mouse WAT, we discovered a *Mipep* splice variant that lacks exons 15 and 16; we designated this splice variant “ΔMIPEP”. In this study, we characterized the molecular features of ΔMIPEP using computer simulation and a supplemental biological approach.

## 2 Materials and methods

### 2.1 Animals and diets

All animal procedures were approved by the Ethics Review Committee for Animal Experimentation at Tokyo University of Science (Approval number: Y20043 and Y20045). Male mice (CLEA Japan, Tokyo, Japan) were housed under specific pathogen-free conditions at 23 °C with a 12-h light/dark cycle in the Laboratory Animal Center, Faculty of Pharmaceutical Sciences, Tokyo University of Science. Mice were provided with water and a CRF-1 diet (Oriental Yeast, Tokyo, Japan). At 37 weeks of age, mice were euthanized by isoflurane inhalation (WAKO, Osaka, Japan), and subcutaneous WAT (sWAT), epididymal WAT (eWAT), brown adipose tissue (BAT), and liver were collected. Tissues were immediately minced, flash-frozen in liquid nitrogen, and stored at −80 °C until analysis.

### 2.2 Cell culture and adipocyte differentiation

3T3-L1 cells were purchased from Japanese Collection of Research Bioresources (Osaka, Japan). They were maintained in low-glucose Dulbecco's modified Eagle's medium (DMEM, WAKO) supplemented with 10 % fetal bovine serum (FBS, Capricorn Scientific, Ebsdorfergrund, Germany, and Nichirei Biosciences Inc., Tokyo, Japan) and 1 % penicillin/streptomycin (Sigma-Aldrich, St. Louis, MO, USA). Human mesenchymal stem cells (hMSCs: SF-87 and SF-89) were kindly provided by BioMimetics Sympathies (Tokyo, Japan) and maintained in high-glucose DMEM supplemented with 10 % FBS and 1 % penicillin/streptomycin. To induce adipocyte differentiation, when hMSCs were cultured to confluency, the culture medium was changed to differentiation medium (high-glucose DMEM containing 1 μM dexamethasone (Sigma-Aldrich), 50 μM indomethacin (WAKO), 1 μM pioglitazone hydrochloride (Tokyo Chemical Industry, Tokyo, Japan), 10 μg/mL insulin (Sigma-Aldrich), and 500 μM 3-isobutyl-1-methylxanthine (Sigma-Aldrich)). The differentiation medium was changed every other day and used for semiquantitative RT-PCR.

### 2.3 CRISPR-Cas9 system

CRISPR-Cas9 system was performed as reported previously [10]. Briefly, the crRNA sequence (5′-AGTACTAGCTGGTCCCCGGT-3′) targeting exon 1 of the *Mipep* gene was designed using the online tool CHOPCHOP (https://chopchop.cbu.uib.no/). Potential off-target effects were evaluated with COSMID (https://crispr.bme.gatech.edu). Designed crRNA and Alt-R® CRISPR-Cas9 tracrRNA-ATTO™ 550 [Integrated DNA Technologies (IDT), Coralville, IA, USA] were annealed to form a duplex in Nuclease Free Duplex Buffer (IDT). The crRNA:tracrRNA duplex was combined with Alt-R® S.p. Cas9 Nuclease 3NLS (IDT) to assemble ribonucleoprotein complexes. 3T3-L1 cells were transfected with the ribonucleoprotein complexes using Lipofectamine® RNAiMAX Transfection Reagent (Thermo Fisher). After 48 h, cells with elevated ATTO™ 550 fluorescence were isolated as single cells using the BD FACS Melody™ II (BD Biosciences, Franklin Lakes, NJ, USA).

### 2.4 Construction of plasmids for full-length *Mipep* or *ΔMipep* overexpression

To construct a full-length *Mipep* overexpression vector, *Mipep* was PCR amplified from mouse eWAT cDNA that was prepared using ReverTra Ace® qPCR RT Master Mix with gDNA Remover (TOYOBO, Osaka, Japan), KOD FX Neo (TOYOBO), and the following primers: forward 5ʹ-GTACGGGAATTCGCCACCATGCTGCTGGCGGCCGG-3ʹ and reverse 5ʹ-CGGCCGCTCGAGCTATTTAGAATCCAGGAAGAACG-3ʹ. The insert was digested with EcoRⅠ/XhoI and then ligated into pBluescript Ⅱ SK(+). The insert sequence of purified plasmid DNA was analyzed by the Sanger sequencing service of Eurofins Genomics (Tokyo, Japan) using the primers shown in Table S1. Each plasmid confirmed to harbor full-length *Mipep* or *ΔMipep* was digested with EcoRⅠ/XhoI to release the inserts. These inserts were then ligated into pMXs-AMNN-Puro or pMXs-AMNN-Neo, respectively [11].

### 2.5 *Mipep* or *ΔMipep* overexpression in 3T3-L1 and *Mipep* KO cells

*Mipep* and *ΔMipep* overexpression was achieved using a retroviral vector based on a previously reported method [11]. Briefly, pMXs-AMNN-puro (mock), pMXs-AMNN-*Mipep*-puro (*Mipep* overexpression) or pMXs-AMNN-*ΔMipep*-puro (*ΔMipep* overexpression) were transfected into Plat-E cells (kindly provided by T. Kitamura, University of Tokyo, Japan) using PEI-MAX® (Polysciences Inc, Warrington, PA, USA). The supernatant collected from each virus-containing culture was added to 3T3-L1 or *Mipep* KO cells, followed by treatment with 2 μg/mL puromycin for 5 days. The 3T3-L1 cells or *Mipep* KO cells infected with the mock-virus were used as control cells. Multiple overexpressing cell clones were independently established for each construct.

### 2.6 Semiquantitative RT-PCR and quantitative real-time RT-PCR (qPCR)

Total RNA was extracted using ISOGENⅡ (Nippon Gene, Tokyo, Japan). cDNA was synthesized from purified RNA using ReverTra Ace® qPCR RT Master Mix with gDNA Remover (TOYOBO). qPCR for *Mipep* was performed using CFX Connect™ (Bio-Rad, Hercules, CA, USA), specific primers, and THUNDERBIRD® SYBR™ qPCR Mix (TOYOBO). qPCR for *ΔMipep* was performed using CFX Connect™, a FAM-TAMRA probe (5′-CAGCTGCCAGGCCTGACCAGTCTG-3′; Takara), specific primers, and THUNDERBIRD Probe qPCR Mix (TOYOBO); these primers were designed/validated by Takara. Reactions followed manufacturer protocols. Plasmids pMXs-AMNN-*Mipep*-Neo and -*ΔMipep*-Neo (8.19 × 10^−7^ and 6.41 × 10^−7^ pmol/μL) were serially diluted ( × 5) for calibration curves. Absolute mRNA levels were calculated as copy numbers per μg total RNA. To detect *ΔMipep* in mouse tissues and assess hMSC adipogenic differentiation, semiquantitative RT-PCR was performed using Blend Taq-Plus (TOYOBO), followed by agarose gel electrophoresis and ethidium bromide staining, analyzed via LAS3000 (Fujifilm, Tokyo, Japan) or ChemiDoc™ (Bio-Rad). hMSC gene expression was normalized to *PPIA*. Primer sequences are shown in Table S2; human primer positions are shown in Supplementary Fig. 1.

### 2.7 Western blotting

Procedures for cell lysis and immunoblotting followed protocols established in our previous works [7,11]. Briefly, following lysis with SDS sample buffer, cells were boiled and sonicated. Proteins (15–30 μg per sample) were separated by SDS-PAGE and transferred to nitrocellulose membranes. Membranes were blocked for 60 min at room temperature using 2.5 % skim milk and 0.25 % BSA in TBS-T, and incubated overnight at 4 °C with the primary antibodies Primary antibodies against mouse MIPEP (produced as stated below), SIRT3 (5490, Cell Signaling Technology (CST), Beverly, MA, USA), MDH2 (8610, CST), COX4 (4844, CST), and LaminB1 (PM064, MBL, Tokyo, Japan) were used. After washing with TBS-T, membranes were incubated with secondary [horseradish peroxidase-conjugated F(ab’)2 fragment of goat anti-rabbit IgG; Jackson ImmunoResearch, West Grove, PA, USA] for 60 min at room temperature. Following additional TTBS washes, signals were developed using ImmunoStar® LD reagent (WAKO) and detected with a ChemiDoc™ Imaging System (Bio-Rad).

### 2.8 Production of anti-mouse MIPEP antibody

Generation of an anti-mouse MIPEP antibody was contracted to Eurofins Genomics (Tokyo, Japan). Briefly, rabbits were immunized with a synthetic peptide corresponding to the C-terminal region of a mouse MIPEP sequence (NH_2_–C + GERYRREMLAHGGG-COOH). After several immunizations with this peptide-based adjuvant, whole blood was collected from the rabbits and antibodies were affinity-purified using immobilized immunogen.

### 2.9 AlphaFold2-based structure prediction

For the generation of MIPEP and ΔMIPEP models, AlphaFold2 v2.3.2 was used with sequences obtained from UniProt [12,13]. The input parameters were: (a) Entry: A6H611; (b) sequence: 34–711 for MIPEP and 34–711 without 549–614 for ΔMIPEP; (c) model types: Alpha-fold2_monomer; (d) for other parameters, the default settings were used. AlphaFold2 was selected rather than AlphaFold3 because recent benchmark analyses demonstrated that AlphaFold2 can still outperform AlphaFold3 *in de novo* prediction of proteins with limited homologous sequences [14].

### 2.10 Molecular dynamics (MD) simulations

Molecular dynamics (MD) simulations were performed using GROMACS 2025.1 with the AMBER ff14SB force field. The AlphaFold2-predicted structures of MIPEP and ΔMIPEP were simulated for 10 ns in explicit solvent. The root mean square deviation (RMSD) and the root mean square fluctuation (RMSF) of Cα atoms were calculated to evaluate the global stability and local flexibility of the proteins.

### 2.11 Docking simulation and refinement

The sites of MDH2 cleaved by MIPEP were predicted by MitoFates (https://mitf.cbrc.pj.aist.go.jp/MitoFates/cgi-bin/top.cgi) [14]. The 15 amino acid residues centered on the cleavage site were identified and used for the docking simulation with the Alphafold2 models of MIPEP and ΔMIPEP. The HPEPDOCK 2.0 server (http://huanglab.phys.hust.edu.cn/hpepdock/) was used with the following parameters: (a) Specified binding site: H:460, E:461, H464, and H:467; and (b) number of peptide conformation: 1000. The top 100 conformations with the highest binding scores were used for further analysis. HPEPDOCK was selected because it has been validated as an efficient and accurate tool for peptide–protein docking [15]. For refinement, binding energies were calculated using Rosetta 3.14 FlexPepDock according to the Rosetta refinement protocol [[16], [17], [18]], as this approach has been widely used to improve docking predictions following CABS-dock [19]. Binding energy was calculated from a single complex with one peptide located closest to the active site of full-length MIPEP or ΔMIPEP. The same analysis was performed for COX4. UCSF ChimeraX was used to create the model representations presented in the figures (https://www.rbvi.ucsf.edu/chimerax) [20].

## 3 Results

**Fig. 1:**
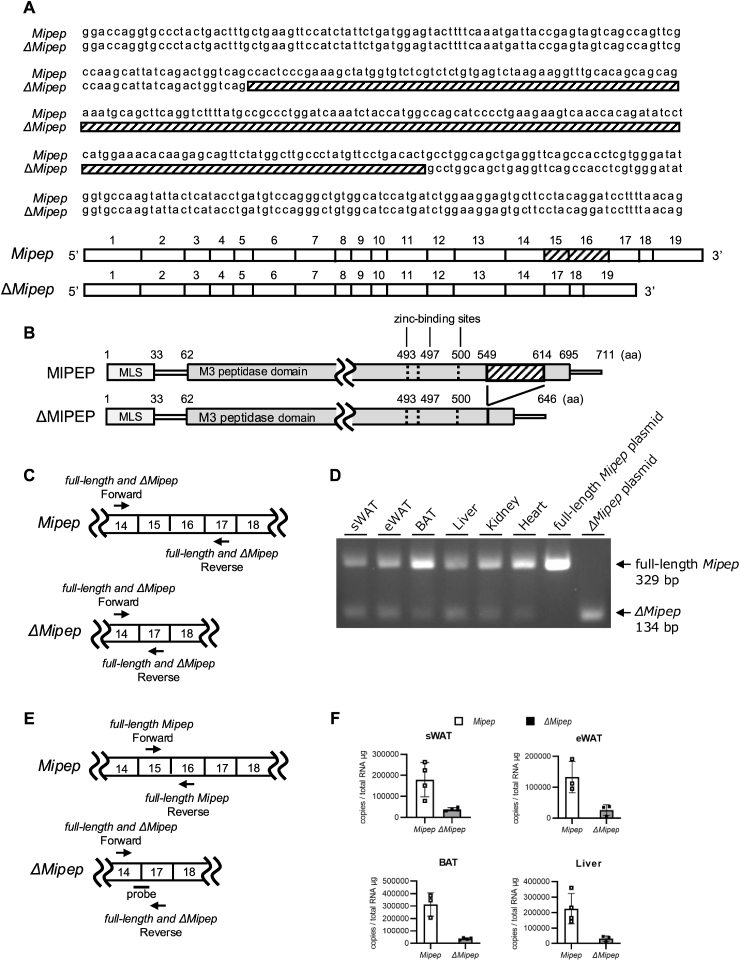
*ΔMipep* is a splice variant of *Mipep*. (A) Sequences from exon 14 to 17 and the exon structures of mouse *Mipep* and *ΔMipep*. The shaded area represents exons 15 and 16. (B) The domain structure of MIPEP and ΔMIPEP. Amino acids 493, 497, and 522 are zinc-binding sites. (C) The positions of primers for semiquantitative RT-PCR analysis of *Mipep* and *ΔMipep* cDNAs. (D) Agarose gel electrophoresis images of semiquantitative RT-PCR for full-length *Mipep* and *ΔMipep* in mouse tissues. Full-length *Mipep* plasmid (pMXs-AMNN-*Mipep*-Puro) and *ΔMipep* plasmid (pMXs-AMNN-*ΔMipep*-puro) were used as positive controls of full-length *Mipep* and *ΔMipep*, respectively. (E) The positions of primers and the probe for quantitative RT-PCR analysis of *Mipep* and *ΔMipep* cDNAs. (F) Copy numbers of full-length *Mipep* and *ΔMipep* mRNAs per total RNA μg in mouse tissues. Values are shown as the mean ± standard deviation (n = 3 or 4).

Sequence analysis of the plasmids containing the amplified *Mipep*-coding sequences derived from cDNA of mouse WAT identified full-length *Mipep* and a *Mipep* splice variant that lacks exons 15 and 16 (*ΔMipep*) (Fig. 1A). This analysis also showed that the M3 peptidase domain and zinc-binding sites (amino acids residues 493, 497, and 500) (registered in UniProt, https://www.uniprot.org/uniprotkb/A6H611/entry) were preserved in ΔMIPEP (Fig. 1B). To validate the expression of endogenous *ΔMipep*, we performed semiquantitative RT-PCR using primers recognizing exon 14 and 17 (Fig. 1C and Table S2, full-length and *ΔMipep*) that can simultaneously detect both full-length *Mipep* and *ΔMipep* in mouse sWAT, eWAT, BAT, liver, kidney, and heart tissues. This analysis detected *ΔMipep* bands (134 bp) in all tissues, the intensities of which were lower than those of full-length *Mipep* bands (329 bp) (Fig. 1D). We then determined the mRNA levels of full-length *Mipep* and *ΔMipep* in several mouse tissues by quantitative RT-PCR using probe-based methods (Fig. 1E). In agreement with the semiquantitative RT-PCR results, *ΔMipep* was expressed in sWAT, eWAT, BAT, and liver, and its levels were lower than those of full-length *Mipep* in all tissues (Fig. 1F). These data support the existence of endogenous *ΔMipep*. *ΔMIPEP*, a human *MIPEP* splice variant that also lacks exons 15 and 16 was detected in hMSCs (Supplementary Fig. 1) We therefore consider *ΔMipep* to be a minor splice variant in mouse and human.

**Fig. 2:**
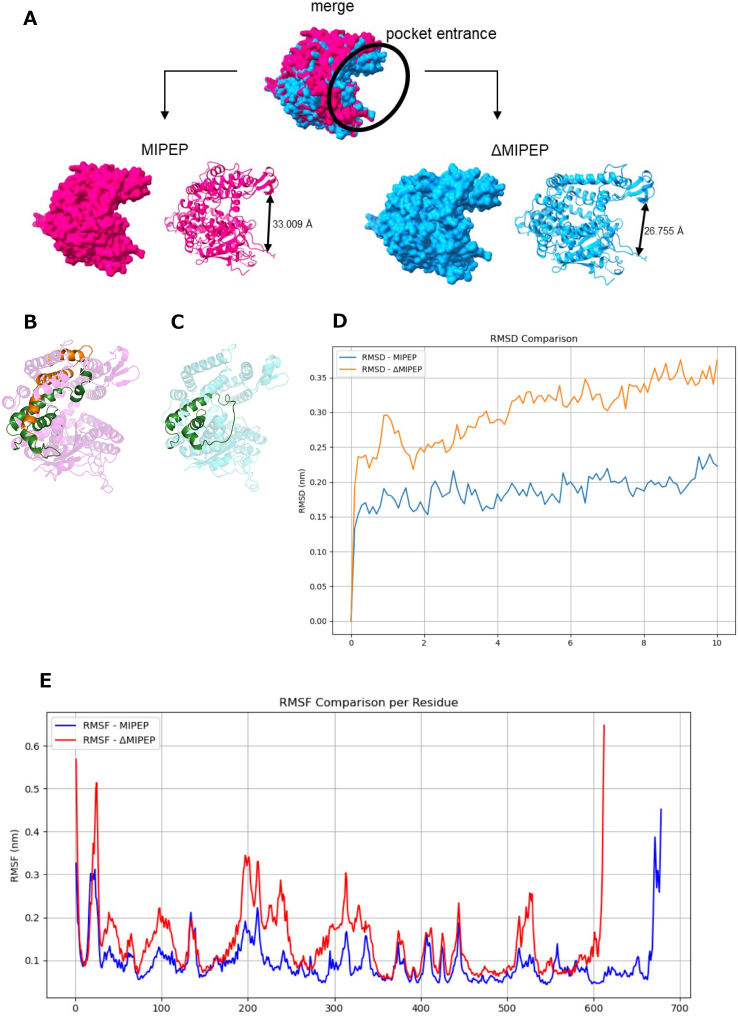
Predicted structures and structural characteristics of MIPEP and ΔMIPEP proteins. (A) Structure prediction of MIPEP (magenta) and ΔMIPEP (blue) proteins using AlphaFold2. The pocket entrance size is shown for each structure. The upper image shows the merged structure of MIPEP and ΔMIPEP. (B) The cartoon representation of MIPEP is shown with specific exons highlighted. Residues 479–515 (exon 14) and 582–622 (exon 17) are colored forest green, while residues 516–581 (exons 15–16) are colored orange to emphasize the spliced region of interest. (C) The cartoon representation of ΔMIPEP (711 residues with deletion of residues 516–581, corresponding to exons 15–16) is shown. Residues 479–556, encompassing exons 14 and 17, are highlighted in forest green to emphasize the continuous region formed after exon 15–16 deletion. (D) The root-mean-square deviation (RMSD) of Cα atoms was calculated during 10 ns molecular dynamics simulations. The RMSD profile of wild-type MIPEP (blue) and ΔMIPEP (orange) is shown as a function of simulation time. The overall stability of the trajectories was assessed based on the convergence of RMSD values. (E) The root-mean-square fluctuation (RMSF) of Cα atoms was computed from the same 10 ns simulations. Per-residue fluctuations are plotted for MIPEP (blue) and ΔMIPEP (red). Peaks indicate flexible regions, whereas valleys correspond to structurally stable domains.

To assess structural differences between MIPEP and ΔMIPEP, we predicted structures of full-length MIPEP and ΔMIPEP using AlphaFold2. The merged structures in Fig. 2 show that ΔMIPEP (blue) has a narrower pocket entrance than full-length MIPEP (magenta) (the area shown as a black circle in Fig. 2). The size of the pocket entrance of ΔMIPEP was calculated to be 26.755 Å, while that of full-length MIPEP was 33.009 Å (Fig. 2). We also visualized the position of exons 15–16 in the protein structure, which connect the upper and lower domains of MIPEP (Fig. 2B and C). To further evaluate the structural flexibility of MIPEP and ΔMIPEP, we performed 10-ns MD simulations. RMSD analysis revealed that MIPEP fluctuated within the range of 0.15–0.25 nm, whereas ΔMIPEP exhibited larger fluctuations of 0.20–0.35 nm, indicating that ΔMIPEP is less structurally stable than MIPEP (Fig. 2D). In addition, RMSF analysis showed that both MIPEP and ΔMIPEP maintained comparable structural stability in the catalytic region (residues 450–500) (Fig. 2E).

**Fig. 3:**
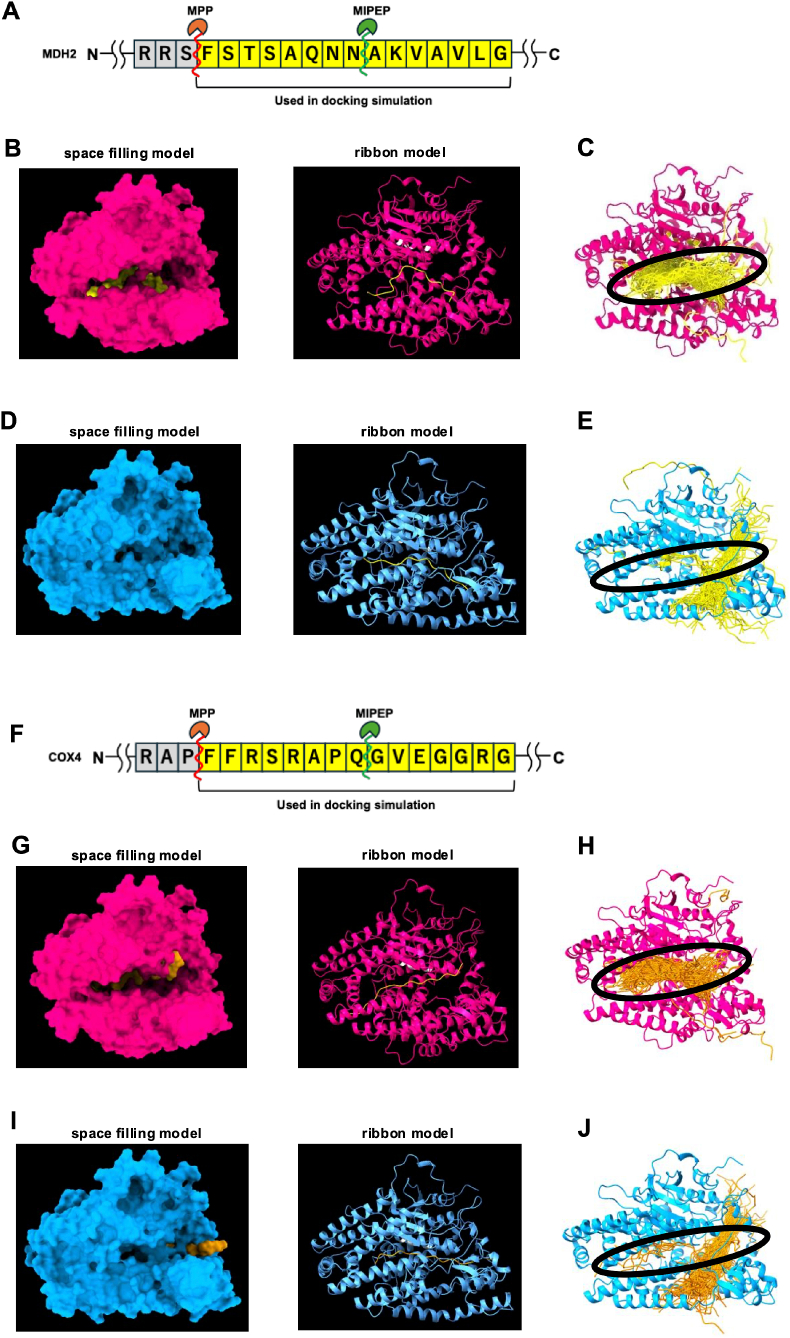
Docking simulation between MIPEP or ΔMIPEP and substrates. (A, F) N-terminal residues of malate dehydrogenase 2 (MDH2) (A) and cytochrome *c* oxidase subunit 4 (COX4) (F). The cleavage sites of MDH2 and COX4 by MIPEP are shown. The yellow area indicates the 15 amino acid residues used for docking simulation. (B, D, G, I) Docking models of MDH2 (B–E) and COX4 (G–J) 15-aa fragments (yellow or orange) with AlphaFold2 models of MIPEP (magenta) and ΔMIPEP (blue), prepared using the HPEPDOCK server. The left images show space filling models, while the right images show ribbon models. The three zinc-binding sites of MIPEP are colored white. (C, E, H, J) The top 100 conformations with the highest scores of each docking simulation, respectively (B, D, G, I).

Given the different-sized pocket entrances, we compared the binding of full-length MIPEP and ΔMIPEP to substrates by performing docking simulations with MDH2, a MIPEP substrate [3]. Based on the substrate cleavage motif of substrates cleaved by Oct1, a yeast ortholog of MIPEP, [RX(↓)(F/L/I)XX(T/S/G)XXXX(↓); arrows indicate MPP and Oct1 cleavage sites, respectively] [21,22], we prepared an MDH2 peptide sequence of 15 amino acids that included a MIPEP cleavage site predicted by MitoFates (https://mitf.cbrc.pj.aist.go.jp/MitoFates/cgi-bin/top.cgi) [23] (Fig. 3A). The docking simulation of full-length MIPEP and this MDH2 peptide showed that the center of the MDH2 peptide sequence was close to the zinc-binding sites, which are known to be important for the enzyme activity of full-length MIPEP. Furthermore, this simulation showed the docking to have low binding energy: −1240.555 cal/mol (Fig. 3B). We repeated the same simulation 100 times and almost all simulations showed that the MDH2 peptide entered the pocket-like structure (the area shown within the black circle in Fig. 3C), indicating that this area is probably the substrate-binding pocket of MIPEP (Fig. 3C). Next, we conducted a docking simulation between ΔMIPEP and the same MDH2 peptide. ΔMIPEP could also dock with the MDH2 peptide, although its binding energy was extremely high: 3642.044 cal/mol (Fig. 3D). Furthermore, the top 100 conformations extracted from 1000 repeat trials with HPEPDOCK in the docking simulation showed that the MDH2 peptide hardly entered the pocket structure of ΔMIPEP (the area shown within the black circle in Fig. 3E), unlike full-length MIPEP (Fig. 3E). We then performed a similar docking simulation with 15 amino acid residues of COX4, another substrate of MIPEP (Fig. 3F). As with MDH2, full-length MIPEP–COX4 docking was achieved with low binding energy: −1241.478 cal/mol, but ΔMIPEP–COX4 docking required higher energy: 2119.499 cal/mol (Fig. 3G–J). These results indicate that ΔMIPEP–MDH2 and ΔMIPEP–COX4 docking rarely occur under physiological conditions.

**Fig. 4:**
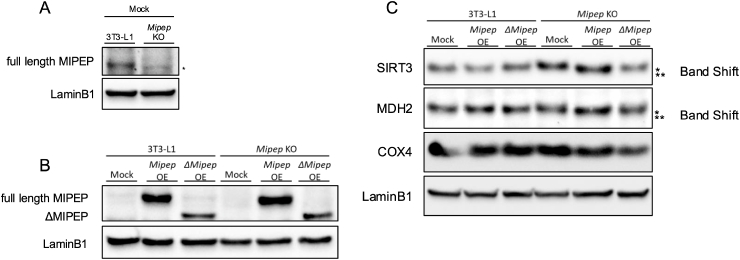
Changes in molecular sizes of MIPEP substrates in *Mipep* knockout cells overexpressing full-length *Mipep* or *ΔMipep.* (A) The lack of MIPEP was confirmed in *Mipep* knockout (KO) cells. (B) Full-length MIPEP and ΔMIPEP protein levels in mock, full-length *Mipep*- and *ΔMipep*-overexpressing 3T3-L1 and *Mipep* KO cells. ∗ indicates a non-specific band. (C) Sirtuin 3 (SIRT3), malate dehydrogenase 2 (MDH2) and cytochrome *c* oxidase 4 (COX4) protein levels in mock, full-length *Mipep*- and *ΔMipep*-overexpressing cells. ∗ indicates the higher molecular weight bands, while ∗∗ indicates the lower molecular weight bands. LaminB1 was used as a loading control.

To investigate differences in full-length MIPEP and ΔMIPEP processing of MIPEP substrates, such as SIRT3, MDH2 and COX4 [3,7], we overexpressed full-length *Mipep* or *ΔMipep* in 3T3-L1 preadipocytes and *Mipep* knockout (KO) 3T3-L1 preadipocytes to avoid the effects of endogenous MIPEP (Fig. 4A and B). Western blotting showed no difference in the levels of overexpressed full-length *Mipep* and *ΔMipep* in 3T3-L1 cells (Fig. 4C). *Mipep* KO-mock cells exhibited SIRT3 and MDH2 bands of slightly higher molecular weights (∗) compared with those in 3T3-L1 cells (∗∗) (Fig. 4C). Moreover, SIRT3 and MDH2 bands were of lower molecular weights in *Mipep* KO-full-length *Mipep* overexpression cells (∗∗) compared with those in *Mipep* KO-mock cells (∗) (Fig. 4C). Notably, *Mipep* KO-*ΔMipep* overexpression cells did not show these band shifts of SIRT3 and MDH2 (Fig. 4C). By contrast, the molecular weight of COX4 bands was constant in all cell lines (Fig. 4C). These changes were also observed in another *Mipep* KO cell clone (Supplementary Fig. 2). These results indicate that ΔMIPEP may not be able to cleave a proportion of a substrate pool.

## 4 Discussion

We have identified ΔMIPEP, a splice variant of MIPEP. As shown in Fig. 2, ΔMIPEP is predicted to have a narrower “substrate pocket” compared with full-length MIPEP. The amino acid residues 549 to 614 that are missing in ΔMIPEP do not include a characteristic domain or the peptidase activity sequence of the MIPEP family (Fig. 1). It is therefore likely that this amino acid sequence contributes to the structure of the protein, rather than affecting its enzyme activity. Consistent with this, visualization of exons 15–16 showed that the deleted regions in MIPEP (Fig. 2B and C) connect the upper and lower parts of the protein. Their absence appears to cause the upper region to adopt a more closed conformation, thereby reducing the overall degree of opening. MD simulations provided further insights into these structural changes. RMSD analysis demonstrated that ΔMIPEP is globally more flexible and structurally less stable than full-length MIPEP (Fig. 2D), whereas RMSF analysis revealed that the catalytic region retained its structural integrity even in the exon 15–16 deletion mutant (Fig. 2E). These findings suggest that the deletion primarily affects the conformational opening of the protein. Although the catalytic region itself remains intact, its accessibility may be restricted in ΔMIPEP. Fig. 3, Fig. 4 show docking simulations between MIPEP or ΔMIPEP and MDH2 or COX4 peptides, which are predicted by MitoFates to contain the MIPEP cleavage site. SIRT3 is also a MIPEP substrate, but docking simulation using the SIRT3 peptide could not be performed because MitoFates could not predict the exact MIPEP cleavage site of the SIRT3 amino acid sequence. The docking simulation indicated that MDH2 and COX4 entered the substrate-binding pocket of full-length MIPEP with low binding energy. In contrast, the binding energies of ΔMIPEP–MDH2 and ΔMIPEP–COX4 were much higher than those of full-length MIPEP with these substrates, which is likely to be because of the narrower substrate pocket of ΔMIPEP. Therefore, in physiological conditions, ΔMIPEP may not be able to recognize MDH2 or COX4 as substrates.

Western blotting showed the molecular weights of SIRT3 bands to be different when full-length *Mipep* or *ΔMipep* were overexpressed in *Mipep* KO cells (Fig. 4C). As mentioned above, MIPEP removes eight amino acid residues from the N-terminus of mitochondrial proteins whose mitochondrial targeting signal has already been cleaved by MPP [2]. Given this function of MIPEP, it is conceivable that the slightly higher molecular weight SIRT3 and MDH2 bands in *Mipep* KO cells represent non-cleaved and immature forms. We also showed that, in *Mipep* KO cells, overexpression of full-length *Mipep* restored the SIRT3 and MDH2 band shifts, whereas overexpression of *ΔMipep* failed to do so (Fig. 4C and Supplementary Fig. 2B). These results indicate that the capacity of ΔMIPEP to recognize the original substrates of MIPEP may be compromised, which might be explained by the narrow substrate-binding site of ΔMIPEP (Fig. 2). Unlike SIRT3 and MDH2, the size of the COX4 band was the same in all cell lines (Fig. 4C), which is inconsistent with the docking simulation results using the COX4 peptide (Fig. 3G–J). The reason for this discrepancy is currently not clear, and therefore requires further investigation.

We identified a human *ΔMIPEP* splice variant, emphasizing the evolutionary significance of ΔMIPEP (Supplementary Fig. 1). Our analysis showed that ΔMIPEP lacks the capacity to recognize MIPEP substrates while retaining the active site of the enzyme. This indicates that ΔMIPEP may be different from a general dominant negative isoform, although the enzymatic activity of ΔMIPEP cannot yet be directly verified due to technical limitations, which is a challenge to be addressed in future studies. A possible explanation of these characteristics is that ΔMIPEP selectively recognizes and cleaves unique substrates. This is supported by the findings of Ailikan et al., who demonstrated that a splice variant of FUBP1 interacting repressor (FIR) is a dominant negative isoform that interacts with proteins other than FIR [24]. Since it is currently difficult to demonstrate in vitro that ΔMIPEP has distinct substrate specificity, further research is needed to identify the ΔMIPEP -specific substrates. However, together, these results indicate that ΔMIPEP may contribute to mitochondrial protein homeostasis in collaboration with full-length MIPEP.

## 5 Conclusions

We have identified a novel MIPEP splice variant, ΔMIPEP. Computer simulation predicts that ΔMIPEP retains the predicted catalytic residues, but its enzymatic function remains to be validated. Additionally, ΔMIPEP fails to recognize and cleave MIPEP substrate proteins, which was partially supported by the experimental data. One limitation of this study is that the molecular weight changes of MIPEP substrates were confirmed only by minor band shifts on western blots, which should be evaluated by different approaches in future research. Further investigations into the biological significance of ΔMIPEP will lead to deeper understanding of MtSPase-mediated regulatory mechanisms of mitochondrial proteins.

## Funding

This work was supported by Grants-in-Aid from the 10.13039/501100001691Japan Society for the Promotion of Science (10.13039/501100001691JSPS) (17H02179, 20H04130, 20KK0228, 17K13231, and 20K19686), the 10.13039/100008732Uehara Memorial Foundation, and the 10.13039/501100002241Japan Science and Technology Agency/Support for Pioneering Research Initiated by the Next Generation (JST 10.13039/501100025019SPRING), Grant Number JPMJSP2151.

## CRediT authorship contribution statement

**Yuina Otani:** Conceptualization, Funding acquisition, Investigation, Writing – original draft, Writing – review & editing. **Michiya Kawarai:** Conceptualization, Investigation. **Masaki Kobayashi:** Conceptualization, Funding acquisition, Methodology, Project administration, Supervision, Writing – review & editing. **Yuka Nozaki:** Conceptualization, Funding acquisition, Writing – review & editing. **Rio Uchida:** Investigation. **Kyo Tezuka:** Investigation. **Miku Yokoyama:** Investigation. **Yuhei Mizunoe:** Funding acquisition. **Takumi Narita:** Methodology. **Hiroshi Haeno:** Methodology. **Kanako Miyano:** Resources. **Yasuhito Uezono:** Resources. **Yoshikazu Higami:** Conceptualization, Funding acquisition, Methodology, Project administration, Supervision.

## Declaration of competing interest

The authors declare that they have no conflict of interest.

## Data Availability

Data will be made available on request.
