# Supplementary figures and images for: Identification of a novel MIPEP splice variant with altered substrate-binding properties
Source: Biochem Biophys Rep. 2025 Oct 29;44:102329. doi: 10.1016/j.bbrep.2025.102329 (PMC12605189; doi:10.1016/j.bbrep.2025.102329)

## Slide 1
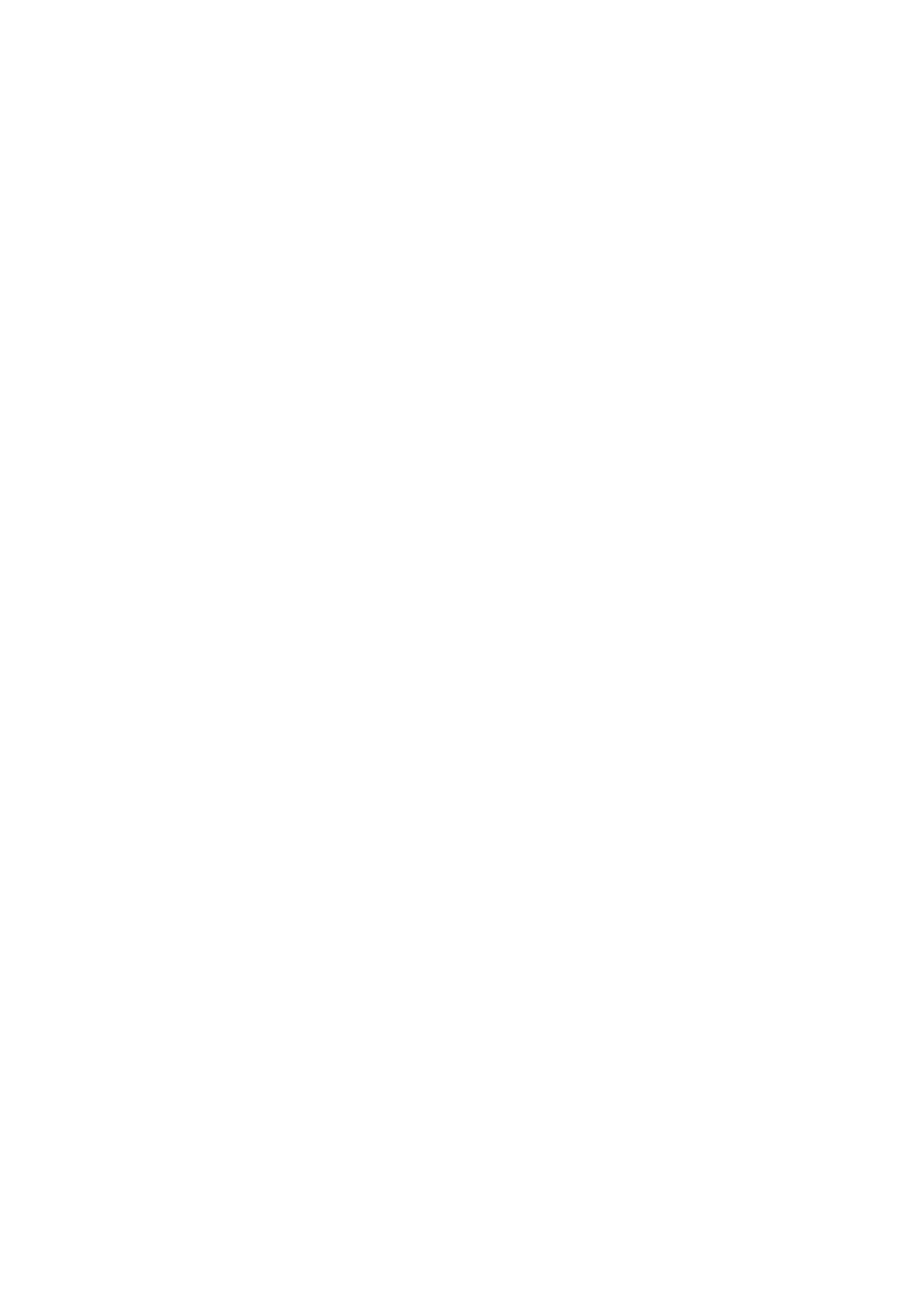

## Slide 2
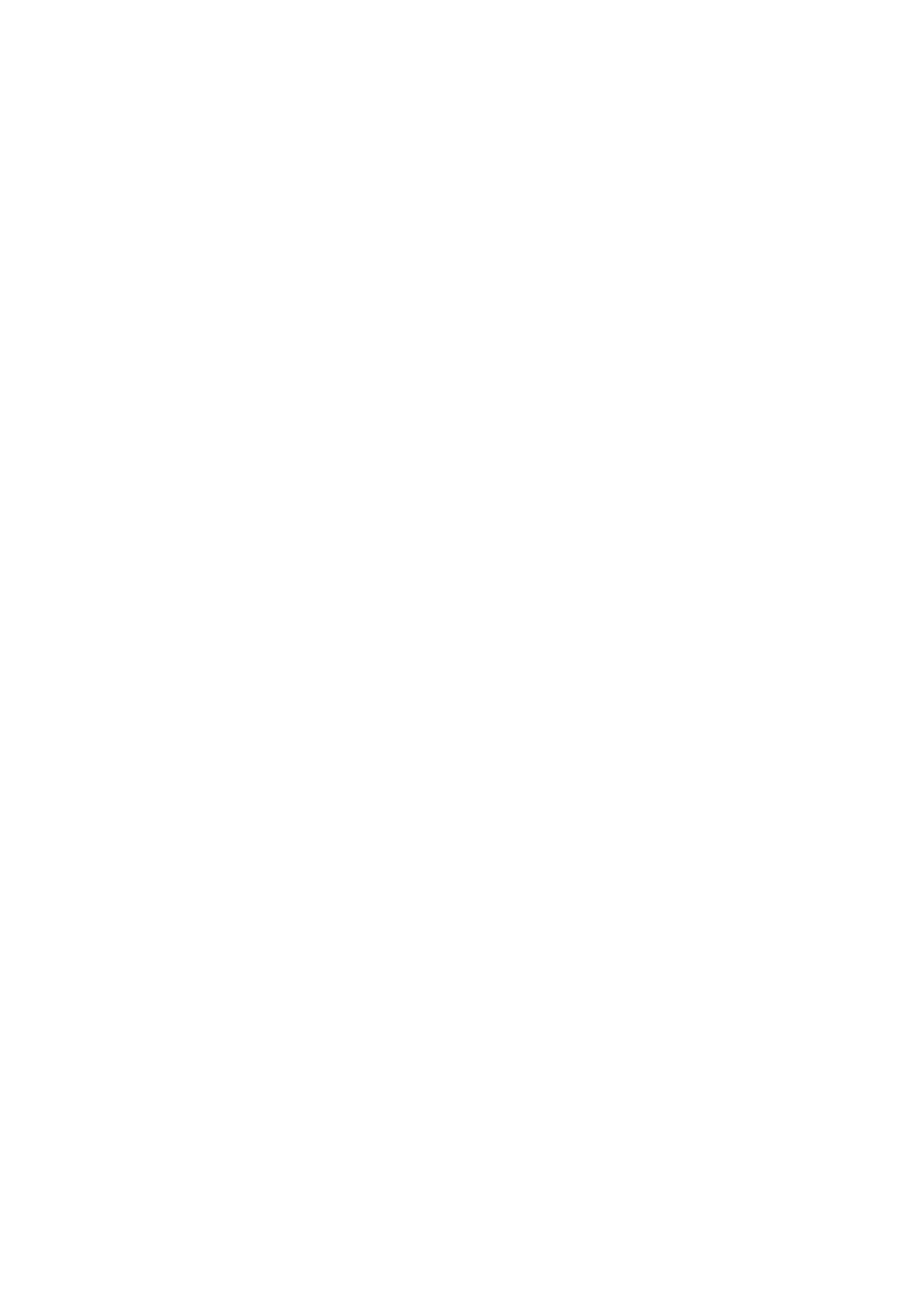

## Slide 3
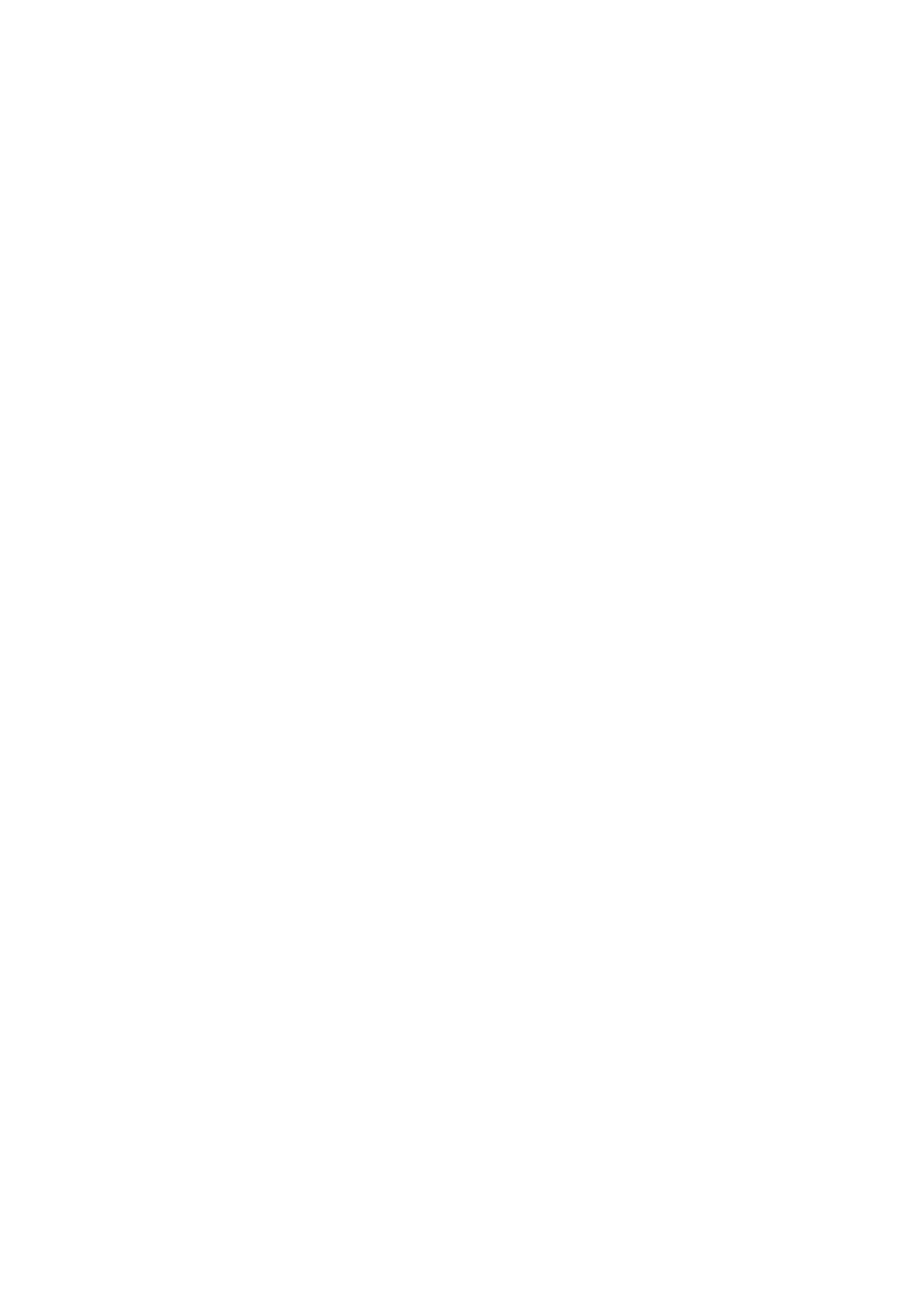

## Slide 4
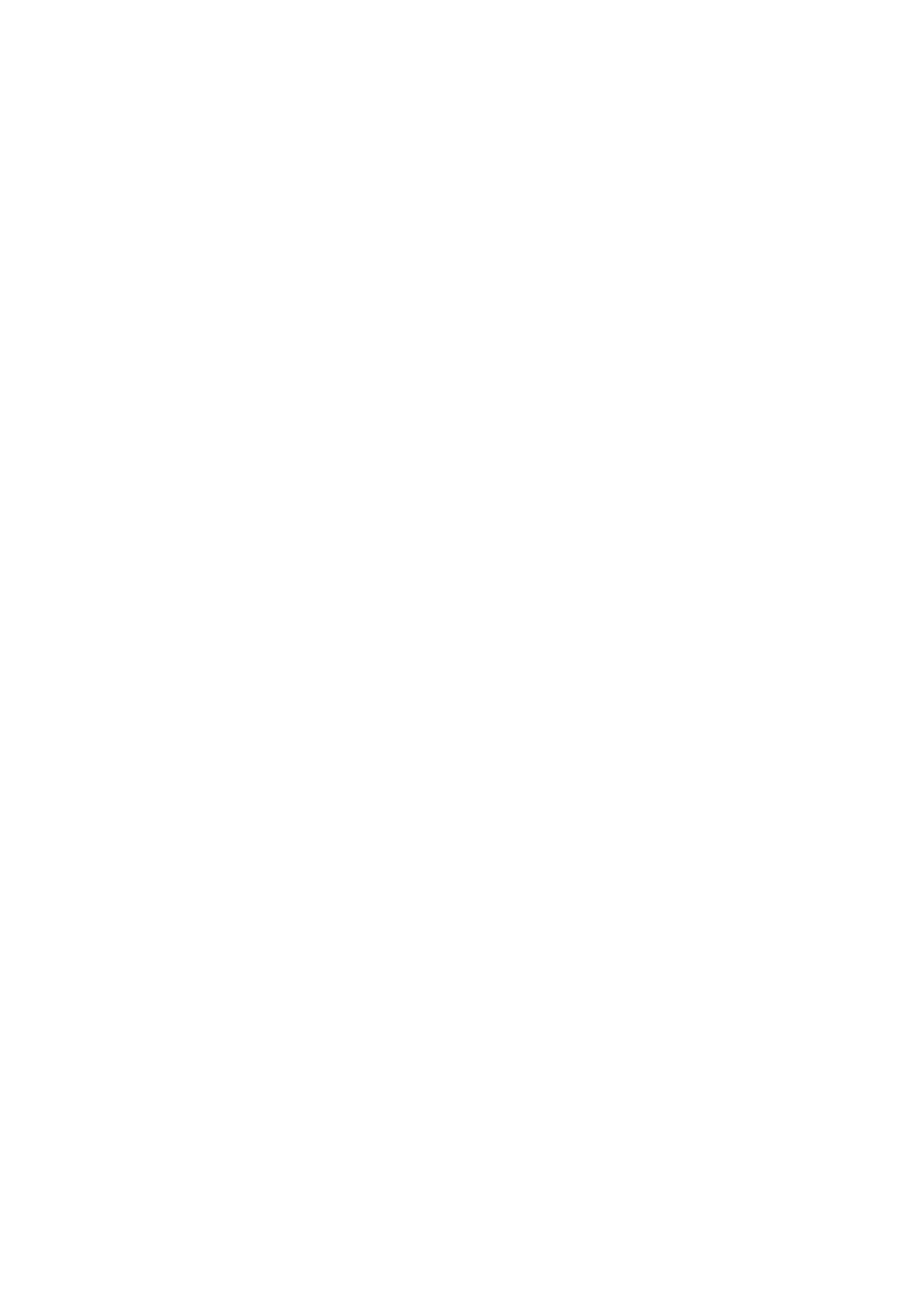

Supplement: Multimedia component 1 [file mmc1.pptx]
