# Supplementary material for: Identification of a novel MIPEP splice variant with altered substrate-binding properties
Source: Biochem Biophys Rep. 2025 Oct 29;44:102329. doi: 10.1016/j.bbrep.2025.102329 (PMC12605189; doi:10.1016/j.bbrep.2025.102329)
Supplement: Multimedia component 2 [file mmc2.pptx]

## Slide 1
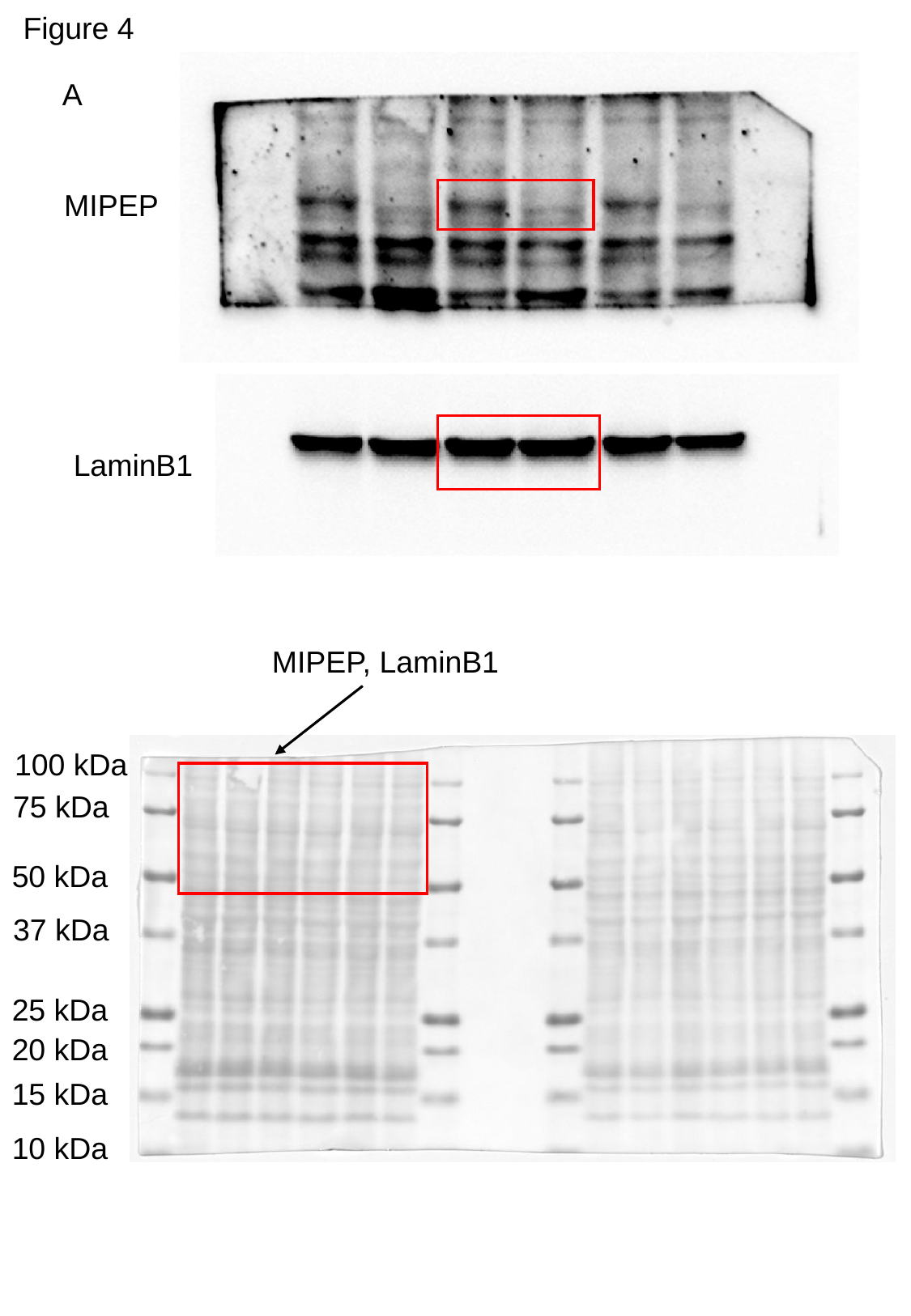

Figure 4
A
MIPEP
LaminB1
MIPEP, LaminB1
100 kDa
75 kDa
50 kDa
37 kDa
25 kDa
20 kDa
15 kDa
10 kDa

## Slide 2
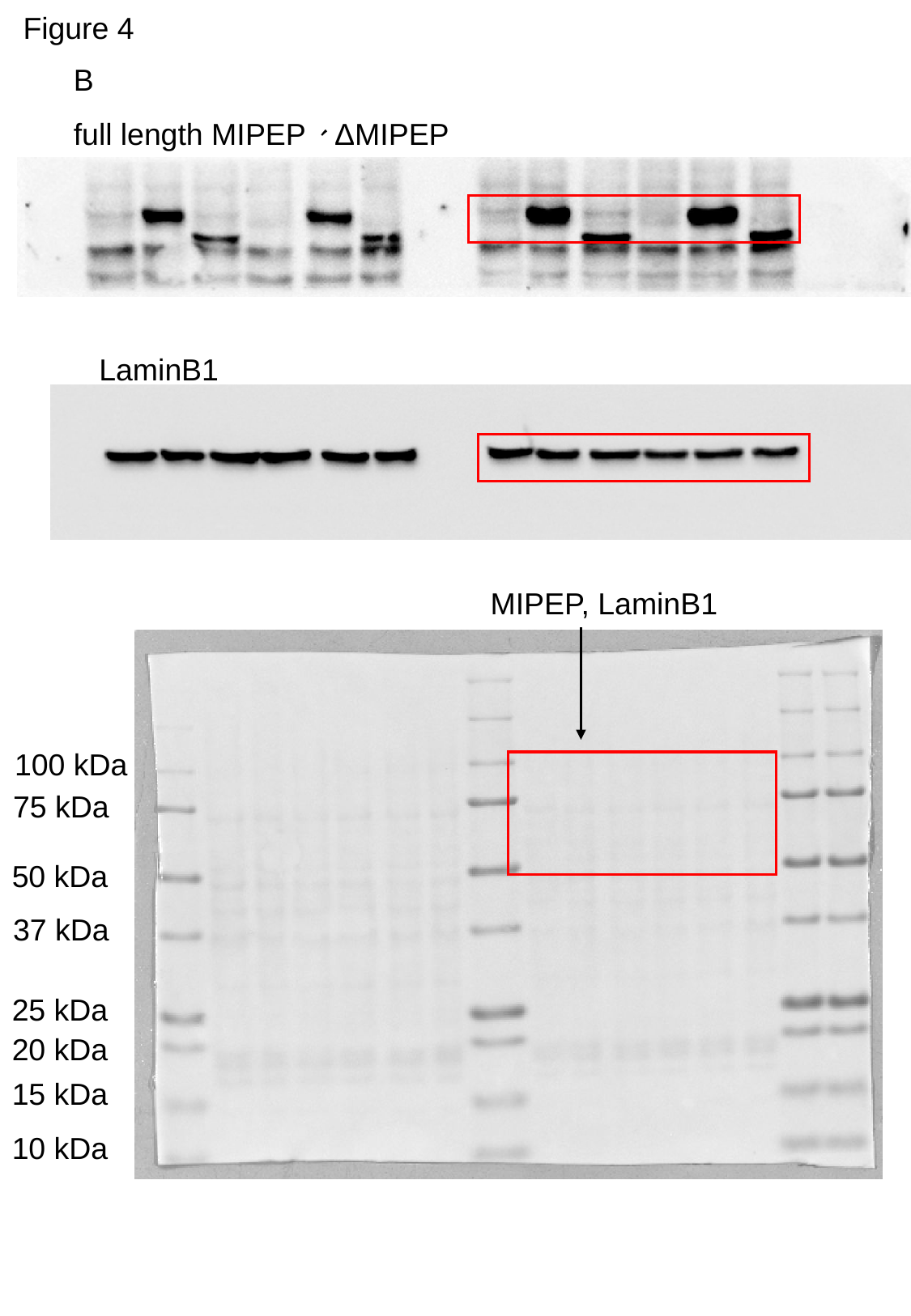

Figure 4
B
full length MIPEP、ΔMIPEP
LaminB1
MIPEP, LaminB1
100 kDa
75 kDa
50 kDa
37 kDa
25 kDa
20 kDa
15 kDa
10 kDa

## Slide 3
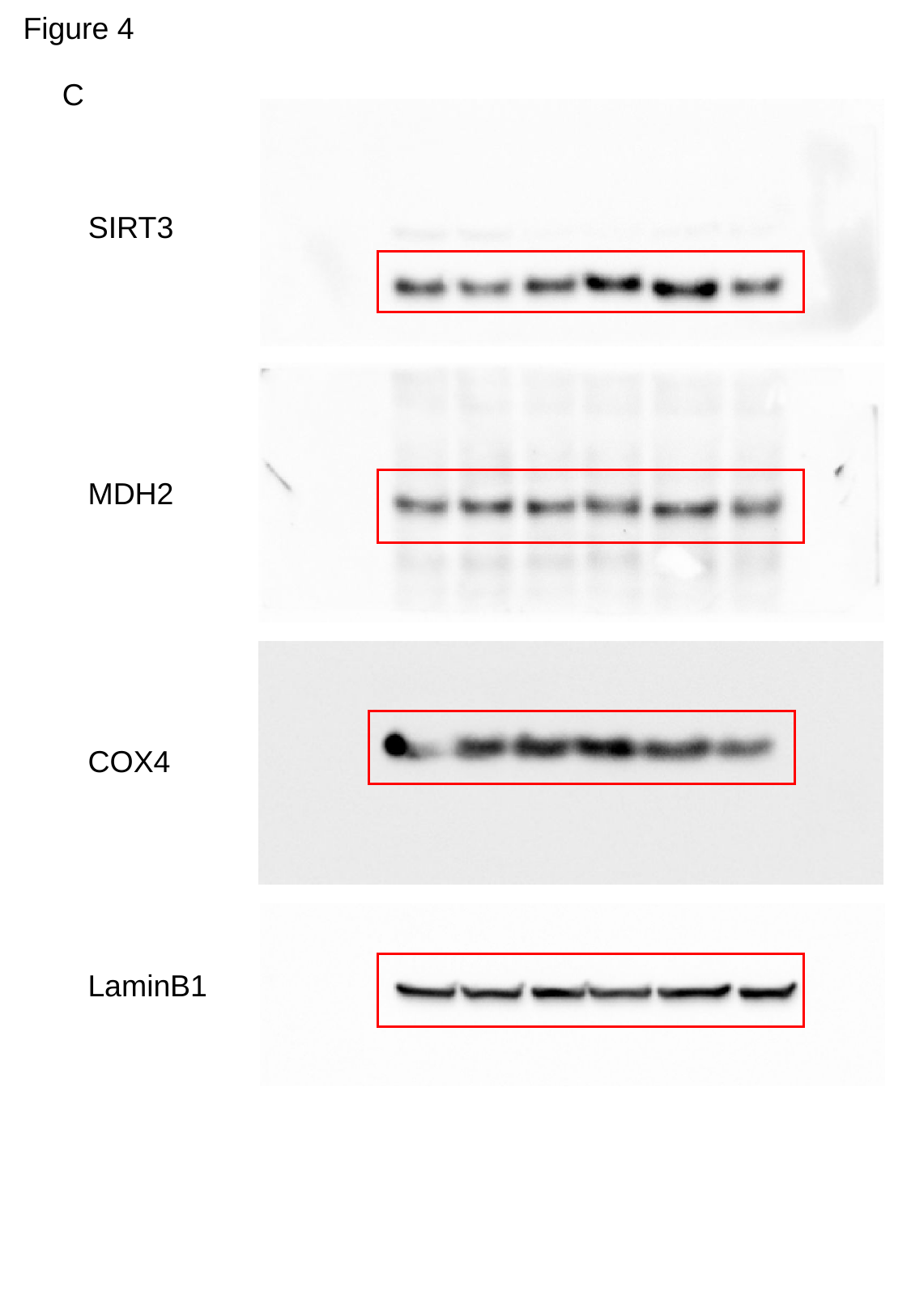

Figure 4
C
SIRT3
MDH2
COX4
LaminB1

## Slide 4
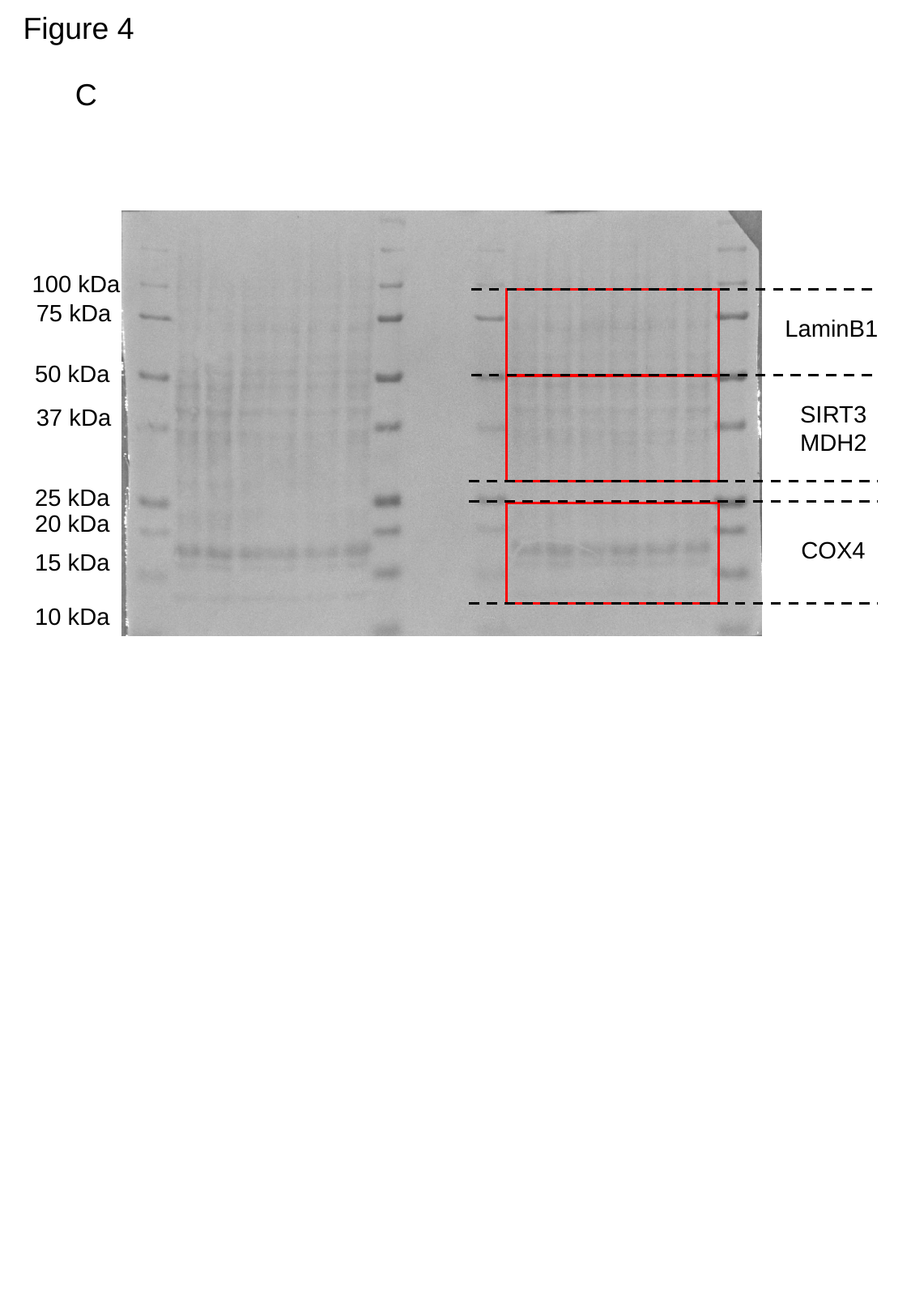

Figure 4
C
100 kDa
75 kDa
LaminB1
50 kDa
SIRT3
MDH2
37 kDa
25 kDa
20 kDa
COX4
15 kDa
10 kDa

## Slide 5
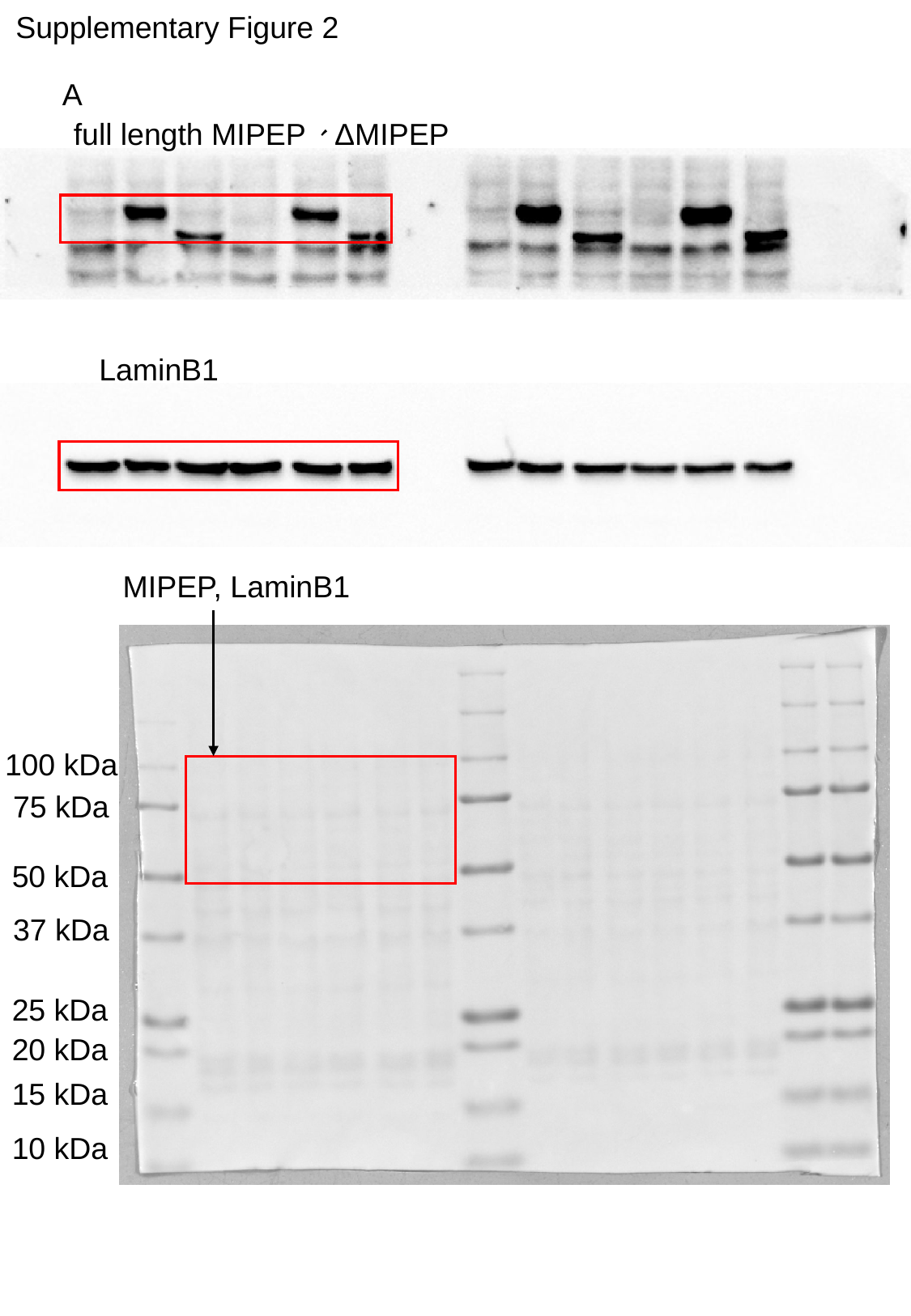

Supplementary Figure 2
A
full length MIPEP、ΔMIPEP
LaminB1
MIPEP, LaminB1
100 kDa
75 kDa
50 kDa
37 kDa
25 kDa
20 kDa
15 kDa
10 kDa

## Slide 6
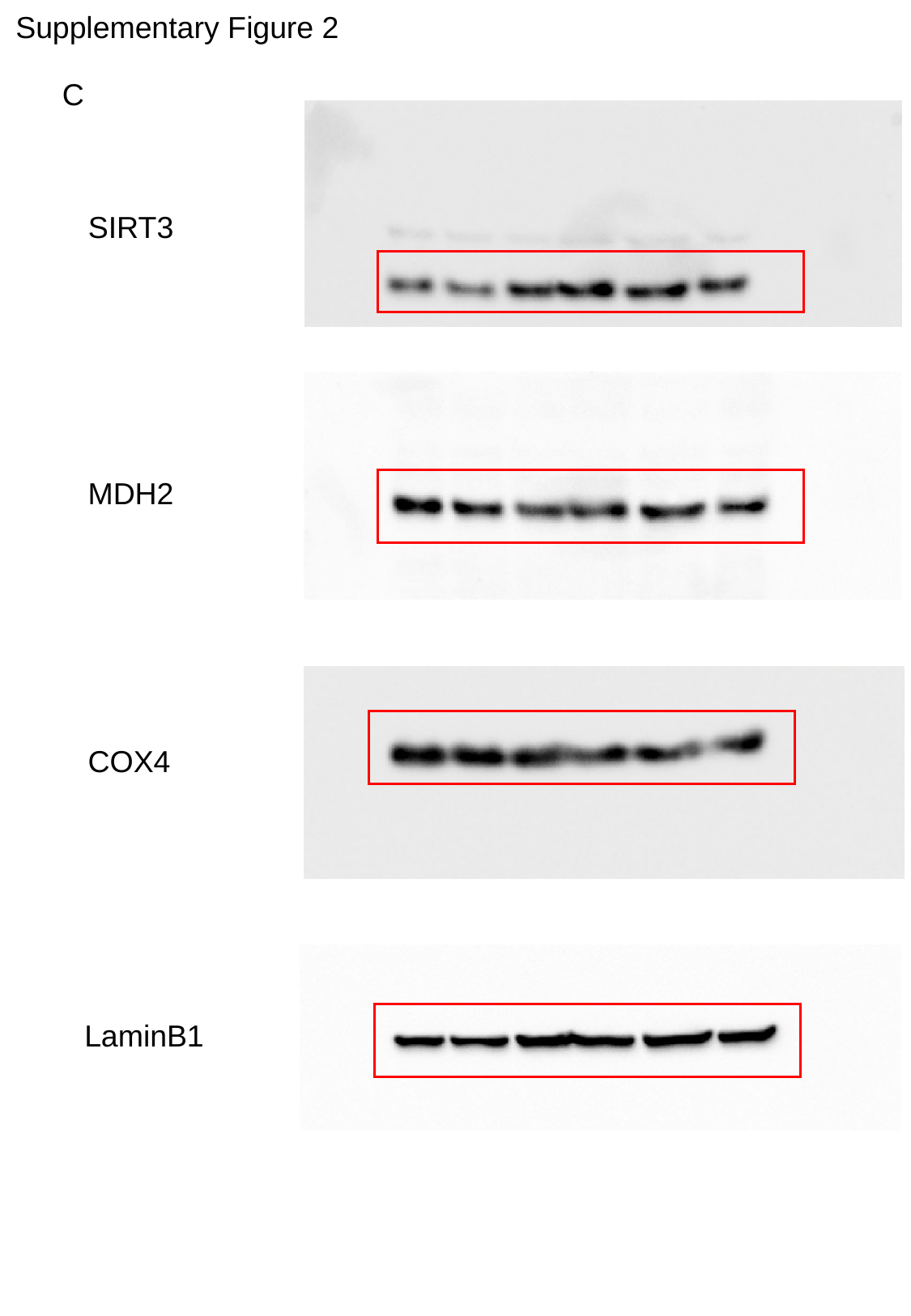

Supplementary Figure 2
C
SIRT3
MDH2
COX4
LaminB1

## Slide 7
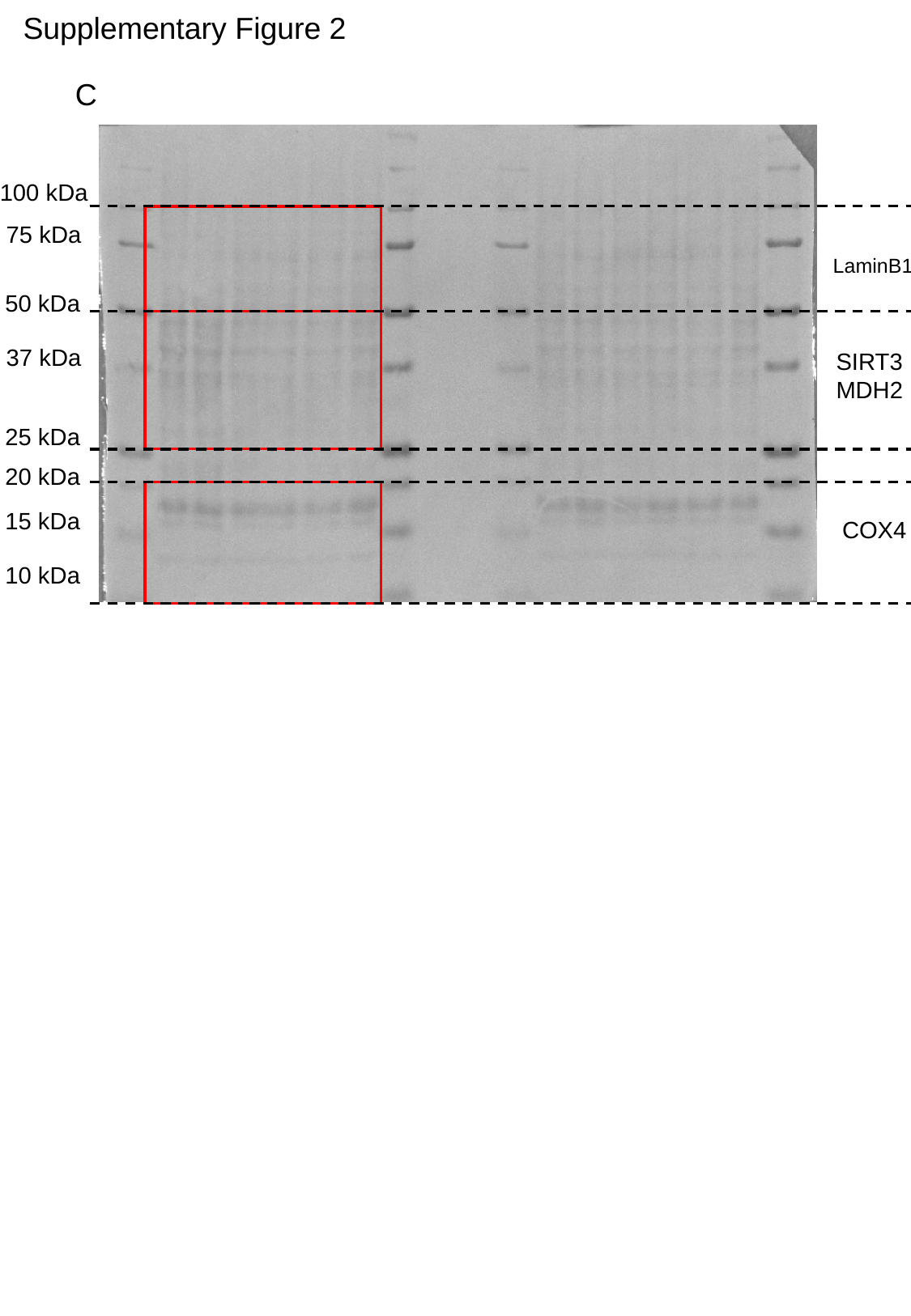

Supplementary Figure 2
C
100 kDa
75 kDa
LaminB1
50 kDa
37 kDa
SIRT3
MDH2
25 kDa
20 kDa
15 kDa
COX4
10 kDa
