# Supplementary material for: Identification of a novel MIPEP splice variant with altered substrate-binding properties
Source: Biochem Biophys Rep. 2025 Oct 29;44:102329. doi: 10.1016/j.bbrep.2025.102329 (PMC12605189; doi:10.1016/j.bbrep.2025.102329)
Supplement: Multimedia component 3 [file mmc3.pdf]

Table S1

M13 Forward	5'-GTAAAACGACGGCCAGT-3'
M13 Reverse	5'-CAGGAAACAGCTATGAC-3'
Mipep_Seq_186	5'-AGAGCCACCTATGGAACCT-3'
Mipep_Seq_449	5'-GAGGCCTGCAGAAGTATTG-3'
Mipep_Seq_731	5'-CTTACCAGAGCACATTCAGC-3'
Mipep_Seq_1035	5'-GATTTCAAGATGATGCAAGG-3'
Mipep_Seq_1342	5'-TTTTCCAGAGAGCAAACAAG-3'
Mipep_Seq_1641	5'-TACCGAGTAGTCAGCCAGTT-3'
Mipep_Seq_1958	5'-ATCCATGATCTGGAAGGAGT-3'

Table S1. List of primers for the sequence analysis of plasmids containing *Mipep* or *ΔMipep*.
