# Supplementary material for: Identification of a novel MIPEP splice variant with altered substrate-binding properties
Source: Biochem Biophys Rep. 2025 Oct 29;44:102329. doi: 10.1016/j.bbrep.2025.102329 (PMC12605189; doi:10.1016/j.bbrep.2025.102329)
Supplement: Multimedia component 4 [file mmc4.pdf]

Table S2

species		Forward<br>Reverse	description
mouse	full-length *Mipep*	5'-GTTTGCACAGCAGCAGAAATG-3'	exon 15
		5'-CTGCTCTTGTGTTTCCATGAGG-3'	exon 16
mouse	full-length and *ΔMipep*	5'-CAGCCAGTTCGCCAAGCA-3'	exon 14
		5'-AGCACTCCTTCCAGATCATGG-3'	exon 17
human	full-length *MIPEP*	5'-TGGCCTACCATATGTTCCAAATAC-3'	exon 16
		5'-CACTTCTGAAGCATACCTTCAACC-3'	exon 18 and 19
human	*ΔMIPEP*	5'-ATTATCAGACTGGACAGGCCTG-3'	junction of exon 14 and 17
		5'-CACTTCTGAAGCATACCTTCAACC-3'	exon 18 and 19
human	*ADIPOQ*	5'-TATGGGGAAGGAGAGCGTAATG-3'	
		5'-CTGAGTTAGTGGTGATCAGTTGGTG-3'	
human	*PPIA*	5'-TACGGGTCCTGGCATCTTGT-3'	
		5'- GATCTTCTTGCTGGTCT-3'	

Table S2. List of primers for qPCR and semiquantitative RT-PCR.
